# Diphtheria

**Published:** 1888-01

**Authors:** L. H. Jones

**Affiliations:** Atlanta, Ga.


### DIPHTHERIA—ITS CAUSE AND TREATMENT.*

BY L. H. JONES, M. D., ATLANTA, GA.

Diphtheria, a disease dreaded alike by the profession and the laity, I shall discuss, first its cause or origin, and then briefly outline what I conceive to be its rational treatment. Diphtheria is a true epidemic disease, to my mind the result of a specific, living and self-reproducing poison. A disease characterized principally by epithelial changes in and the exudation of fibrin on and beneath the mucous membrane of the pharynx, not necessarily originating in or confined to the pharynx, but most usually selecting that as its origin and seat. Now, turning for a moment to our anatomy, we find that the sub-mucous tissue of the pharynx consists of a loose connective tissue, highly vascular, and readily permitting the congestion of the blood-vessels and the extravasation of the corpuscular elements; the epithelium of the mouth and fauces is squamous and not readily detached, while that of the trachea is columnar and ciliated and readily thrown off. We may then, in this anatomical difference in the parts, see some reason why a membrane formed in the pharynx should be harder to detach than one formed in the trachea.

Time will not allow me to go into even the modern history of diphtheria, though Dr. Jacobi, in his article on diphtheria, says: "If the history of any disease is interesting, and the neglect of its study has ever punished itself, it is diphtheria."

Upon our conception and belief as to the origin and cause of the disease, will depend our treatment. I believe it best and safest, certainly most satisfactory to the physician, for us to have firm convictions, and to hold to them, meanwhile striving for more light, and being ever ready to accept new truths when they have been proven to be truths. Probably the most thorough and elaborate study that has been made as to the cause and origin of diphtheria, was done by Drs. Wood and Formad, of Philadelphia.

*A paper read before the Atlanta Society of Medicine.

The result of their studies was embodied in a memoir on the nature of diphtheria, published in 1883. I shall quote largely from this memoir, and by comparing the statements made in the earlier period of their studies with the conclusion reached at the close of their labors, you will see that they were free from bias or preconceived ideas, and that their conclusions were the outgrowth of a careful and diligent study, covering several years of time and including numerous studies with the microscope, both in the laboratory and at the bed-side.

In supplement 7 of the National Board of Health Bulletin, 1880, Drs. Wood and Formad says "it is altogether improbable that bacteria have any direct function in diphtheria."

Again in supplement 17, January, 1882, they say "there is no proof as yet that the micrococci are the cause of the disease."

After this they say: "The obvious conclusion is, that whilst the clinico-pathological evidence does not prove that micrococci are essential to diphtheria, it leads toward such a view and at least shows that some close connection exists between these organisms and the disease."

They found, that as a rule, in all fatal and in many favorable cases the quantity of white blood corpuscles was increased, the average proportion of white to red was frequently about 1-50 and occasionally 1-20.

In twenty-one cases in which the blood was examined, the following statistics are given: In eleven of the cases no micrococci were found in the blood, and only one death, which was the result of laryngeal stenosis. Of the ten cases in which micrococci were found in the blood, six died. In all twenty-one cases the micrococci were found in the membrane.

The micrococci were cultivated in various mediums and were found to flourish best at a temperature of about 37° C.; 98¾ F., but they were not killed by a temperature of 70° C., 158° F. They seemed to need but little oxygen, and thus they seem peculiarly fitted to flourish in dense membranes and deep tissues where the suppply of oxygen must necessarily be small.

The cultivated micrococci seemed to do well up to the fourth generation, when they began to grow weak. But of twenty rabbits inoculated with matter from diphtheritic patients, thirteen died and showed abundant micrococci in the blood and various organs. The blood of rabbits thus injected was taken and injected into other rabbits, and diphtheria resulted. Again, the clear urine of a patient suffering with diphtheria, was filtered and the filter paper then washed and dried; it was found to contain numerous micrococci, and this paper was equally as poisonous as the diphtheritic membrane.

In concluding, Drs. Wood and Formad state that "micrococci are an essential part of the diphtheritic process ; that it is possible to produce diphtheria in animals by inoculation with membrane, pieces of internal organs, urine, blood, etc., taken from persons suffering with diphtheria. That the poison of diphtheria is solid and particulate; that it is found in the urine when the only solid particles present are micrococci, which have grown entirely away from any animal organism." They found that boiling for five minutes did not kill the germs, but that boiling for fifteen minutes did destroy them.

Dr. Wood considers it absolutely certain that diphtheria is contagious, and that the micrococci are capable of inflaming a delicate throat in which no abrasion exists.

Dr. Jacobi, while not conceding the germ origin of diphtheria, says: "As a general thing diphtheria must be looked upon as a constitutional disease, giving rise to local pneumonia in the same way as scarlatina does on the skin, the mucous membrane of the alimentary canal and in the uriniferous tubules; measles on the skin and respiratory mucous membrane; or typhoid in the lymph follicles and on the mucous membrane of the intestines, or in other words, the diphtheritic poison may enter the system locally through a defective or sore or wounded integument, or through the lungs."

That diphtheria is seen most frequently in children may be accounted for by the comparative delicacy of the mucous membrane of the pharnyx and the greater liability of taking cold.

Assuming, then, that micrococci are the cause of diphtheria, the question arises, where do the micrococci come from ? We know that micrococci exist and appear to do best in atmospheres where there are heat, moisture and decomposing organic matter. Just these very conditions are in the sewers of great cities, and it is in cities that we most often find diphtheria originating. The sewer-pipes are usually only partially filled with liquid. Above this liquid, in the upper halves of the pipes, are the gaseous products of decomposing organic matter, capable of supplying the germs of a half dozen different types of disease. The pipes, in order to afford drainage, must slope away from the houses, and warm gases rise naturally towards them The traps in sewer-pipes are rarely perfect, and many systems of sewer-pipes have the waste-pipes from bath-tubs, wash-basins, suits, etc., entering directly and without a trap into the pipe carrying the waste from the bowl of the water closet. Many houses have basins or stationary wash-stands in the sleeping apartments, and thus the occupants of the house sleep in an atmosphere abounding, possibly, in the germs of our most dreaded diseases. Such an arrangement I believe to be not only a fertile source of diphtheria, but also of typhoid fever and various like affections.

No doubt, also, that polluted water may in many epidemics furnish an ample supply of germs.

Omitting the diagnosis, let us now glance at the treatment.

All of the successful treatments of diphtheria are based upon the use of either sulphur or chlorine in some form; and with this idea in view, we find figuring prominently in prescriptions for diptheria the Tr. chloride of iron and the chlorate of potash. But we do no know if the cl. in these compounds is readily set free in the blood, in fact we do not know what chemical changes take place in them when once they have entered the circulation. We do know that they must be given in large quantities and often repeated, if we obtain good results from their use. I believe the good obtained from such remedies is due wholly to the fact that they contain agents which we know are destructive to bacteria, but I also believe that we can use the agents in forms far preferable to those mentioned. If without injury to our patient we can introduce sulphurous acid or free chlorine into the blood, I see no reason why we cannot destroy the micrococci, arrest their further development and cut short the course of the disease. This

I believe we can do, either by the use of the sulphite of soda or chlorine, if we can get sufficient quantity of the remedy into the blood before the micrococci have multiplied too greatly or done too great damage to the blood.

The sulphite of soda readily gives up its sulphurous acid a substance recognized and employed daily as a destroyer of various bacteria.

The question now arises, does the sulphite of soda so break up in the blood as to yield sulphurous acid. Rabateau claimed that it did not, and wrote an elaborate article in support of his belief. More recent experiments have proven the contrary, and sulphurous acid is found in the urine of patients taking sulphite of soda, six hours after taking a dose. Here, then, I believe we have not a specific, but a remedy, which, if employed in time, will cut short the disease, and which we can safely use as a prophylactic, and by its use prevent the spread, not only of diphtheria, but also of scarlet fever, and possibly other contagious diseases. The remedy is harmless, and may be given in doses of ten grs. or more every three or four hours. It may cause the bowels to act too freely, in which case we can diminish the dose or control the bowels by other remedies.

In cases where we may have been called in late, or where the disease assumes a malignant type, I believe we have a more potent agent in chlorine, given as follows:

Take chlorate of potash ʒij, powder finely, and put into a twelve or fourteen ounce bottle; to this add ʒss of hydrochloric acid, and then stopper the bottle. The acid coming in contact with the chlorate of potash, chlorine gas is liberated and fills the bottle. Now pour into the bottle ℥ii. of glycerine and replace the stopper quickly and agitate, thus allowing the glycerine to take up the chlorine. Then fill the bottle with water and cork tightly. We now have the chlorine in solution, and can give from one to two teaspoonful of the mixture, p. r. n. This I believe will rarely fail to destroy the micrococci, even though they be quite numerous. The chlorine is a powerful heart stimulant, and must be given with some caution, and would probably be unsafe as a prophylaxis, and we hold it in reserve for use in such cases as indicated before.

Besides these two agents, the pharmacopeia abounds in drugs which may prove equal or superior to either of those selected.

Besides this special treatment, designed to attack the micrococci, we must remember that diphtheria is a disease especially characterized by great constitutional depression, and the patient should be stimulated early and freely. For this purpose good whisky or brandy is preferable, and may be given in considerable quantities. We should also make use of such local applications to the throat, in the form of sprays, as may seem most beneficial.

Tracheotomy, or intubation, may be resorted to by those so circumstanced as to avail themselves of them.

